# Genome-wide analysis of codon usage bias in four sequenced cotton species

**DOI:** 10.1371/journal.pone.0194372

**Published:** 2018-03-27

**Authors:** Liyuan Wang, Huixian Xing, Yanchao Yuan, Xianlin Wang, Muhammad Saeed, Jincai Tao, Wei Feng, Guihua Zhang, Xianliang Song, Xuezhen Sun

**Affiliations:** 1 State Key Laboratory of Crop Biology/Agronomy College, Shandong Agricultural University, Taian, China; 2 Department of Botany, Government College University, Faisalabad, Pakistan; 3 Heze Academy of Agricultural Sciences, Heze, China; USDA-ARS Southern Regional Research Center, UNITED STATES

## Abstract

Codon usage bias (CUB) is an important evolutionary feature in a genome which provides important information for studying organism evolution, gene function and exogenous gene expression. The CUB and its shaping factors in the nuclear genomes of four sequenced cotton species, *G*. *arboreum* (A_2_), *G*. *raimondii* (D_5_), *G*. *hirsutum* (AD_1_) and *G*. *barbadense* (AD_2_) were analyzed in the present study. The effective number of codons (ENC) analysis showed the CUB was weak in these four species and the four subgenomes of the two tetraploids. Codon composition analysis revealed these four species preferred to use pyrimidine-rich codons more frequently than purine-rich codons. Correlation analysis indicated that the base content at the third position of codons affect the degree of codon preference. PR2-bias plot and ENC-plot analyses revealed that the CUB patterns in these genomes and subgenomes were influenced by combined effects of translational selection, directional mutation and other factors. The translational selection (P2) analysis results, together with the non-significant correlation between GC12 and GC3, further revealed that translational selection played the dominant role over mutation pressure in the codon usage bias. Through relative synonymous codon usage (RSCU) analysis, we detected 25 high frequency codons preferred to end with T or A, and 31 low frequency codons inclined to end with C or G in these four species and four subgenomes. Finally, 19 to 26 optimal codons with 19 common ones were determined for each species and subgenomes, which preferred to end with A or T. We concluded that the codon usage bias was weak and the translation selection was the main shaping factor in nuclear genes of these four cotton genomes and four subgenomes.

## Introduction

Genetic information is transmitted from DNA to mRNA, then from mRNA to protein. In the latter process, information is transmitted in the form of codons. Codon is an important link in the output of nucleic acid information. Genetic code has degenerate feature that a single amino acid, except methionine (Met) and tryptophan (Trp), is encoded by more than one codon known as synonymous codons. The synonymous codons are not used at equal frequencies in coding sequences in many organisms [1]. This phenomenon called ‘synonymous codon usage bias (SCUB)’ reflects non-uniform usage of synonymous codons encoding the same amino acid during the translation of genes to proteins [2,3].

The degree of SCUB divergence varies greatly among different species and genes [4–7]. SCUB was affected by many factors, including directional mutation, neutral selection [8], GC content, synonymous substitution rate [9], tRNA abundance [10], selection for efficient translation initiation [11], codon hydropathy and DNA replication initiation site [12], gene length [13] and expression level [14], etc. Of these factors, directional mutation and neutral selection are the two main ones, with varying relative importance in different species [15–17]. The codon usage patterns always represent balance between the directional mutation and the neutral selection that leads to translational efficiency of genes [8–11]. SCUB patterns were also associated with phylogenetic relationship among given species. Distant phylogenetic species usually has greater variations in codon usage bias [18,19].

Information on the SCUB patterns can provide significant insights pertaining to the prediction, classification, and molecular evolution of genes, design of highly expressed genes and cloning vectors and reveal about the host-pathogen coevolution and adaptation of pathogens to specific hosts [20,21]. Clustering results based on relative synonymous codon usage (RSCU) values could provide useful reference for phylogenetic relationship analysis [18,19,22].

Cotton (*Gossypium* spp.) is the main source of renewable textile fibers and is also grown to produce vegetable oil and high protein meals for humans and livestock [23]. The genus *Gossypium* includes around 46 diploid (2n = 2x = 26) and 6 tetraploid (2n = 4x = 52) and 1 purported species [24–26], including four commercial ones, *G*. *arboreum* (A_2_), *G*. *herbaceum* (A_1_), *G*. *hirsutum* (AD_1_) and *G*. *barbadense* (AD_2_). It has been proposed that all diploid cotton species may have evolved from a common ancestor and allopolyploid cotton may have appeared through hybridization and subsequent polyploidization events between the A- and D-subgenome progenitors. The D-genome species *G*. *raimondii* (D_5_) and the A-genome species much like modern *G*. *arboreum* (A_2_) and *G*. *herbaceum* (A_2_) are the donor species for the D and A chromosome groups of the tetraploid cotton species, respectively [27–28]. Recently, the nuclear genome sequences of *G*. *arboreum*, *G*. *raimondii*, *G*. *hirsutum* and *G*. *barbadense*, and chloroplast and mitochondrial genome sequences of *G*. *hirsutum* have successively been released [29–38], which has advanced the understanding of cotton genomics and genetics, and made it possible to investigate SCUB patterns in cotton nuclear and organelle genomes. But until now, SCUB analysis has been performed only in chloroplast genome of *G*. *hirsutum* [36]. The purpose of this study was to analyze the SCUB patterns of the four sequenced cotton species and explore the key factors influencing codon choice.

## Materials and methods

### Coding sequence data

All the CDS sequences of *G*. *raimondii* [30], *G*. *arboreum* [31], *G*. *hirsutum* [33] and *G*. *barbadense* [34] were downloaded from the CottonGen database (https://www.cottongen.org/). We got the Excel files containing all genes of *G*. *hirsutum* and *G*. *barbadense* from the Cotton Research Institute (CRI) of Nanjing Agricultural University (http://mascotton.njau.edu.cn), and then separated the two allotetraploids into *At* and *Dt* subgenomes using Seqkit [39] (https://github.com/shenwei356/seqkit), named *At*_*1*_ and *Dt*_*1*_ in *G*. *hirsutum*, and *At*_*2*_ and *Dt*_*2*_ in *G*. *barbadense* respectively. Detailed information of CDSs and codons used in this work was listed in Table 1.

**Table 1:** The number of CDSs and codons of 4 cotton species and 4 subgenomes used in this study.

Species or subgenomes	Genome	Number of CDSs	Number of Codons
*G*. *arboreum*	A_2_	40134	14538888
*G*. *raimondii*	D_5_	77267	32995450
*G*. *hirsutum*	(AD)_1_	66434	27096230
*G*. *barbadense*	(AD)_2_	77358	29259373
*At*_*1*_	(AD)_1_	32032	13188672
*Dt*_*1*_	(AD)_1_	34402	13907558
*At*_*2*_	(AD)_2_	39568	14786710
*Dt*_*2*_	(AD)_2_	37790	14472663

### Statistical analyses

#### Codon usage bias indices

The nuclear genome CDSs of each cotton species were firstly analyzed as a whole to clarify the codon usage features. Methionine (Met) and tryptophan (Trp), each having a single codon, were excluded from further analysis. Stop codons (UAG, UAA, and UGA) were also excluded from the analysis because each stop codon can only occur once in a single CDS sequence.

Using CodonW 1.4.2 software (http://codonw.sourceforge.net/), a number of indices of codon usage bias including the relative synonymous codon usage (RSCU), effective number of codons (ENC), and the frequency of the nucleotides G+C at the third position (GC3s) were calculated. Several codon composition indices including GC contents of the entire gene (GC), the content or frequency of each individual base A, T, G, and C at the third position of codons (A3s, T3s, G3s, C3s) were also counted. The G+C content at the first, second positions of codons (GC1, GC2) and the average GC content of the first and second positions (GC12) were determined by the online Cusp program from Galaxy (https://usegalaxy.org/). The correlations between nucleotide contents were calculated with the statistical software SPSS V21.0 (http://www.spss.com.cn/).

#### Multiple comparisons

Multiple comparisons can be used to infer any significant differences between the effects of the factors. Then the T3s, G3s, GC and ENC of each gene were calculated by CodonW 1.4.2. The average value of all the genes was expressed in T3s(av), G3s(av), GC(av) and ENC(av), and multiple comparisons across the cotton species and subgenomes were made by SPSS V21.0. The box and whisker plots were drawn through OmicShare (www.omicshare.com/tools).

#### PR2-bias plot

Parity Rule 2 (PR2) is a rule of DNA composition. When there is no deviation between mutation and selection pressure of two DNA chains, the fractional content of the four bases follows A = T and G = C (where A + T + G + C = 1) [40]. PR2-bias plots are particularly informative when PR2 biases at the third codon position are plotted. The center of the plot, where both coordinates are 0.5, is the place where A = T and G = C (PR2). The degree of deviation from PR2 allows us to estimate the chain bias affected by mutation, selection, or both [41]. If genes are evenly distributed across the plan view, that is, if the codon usage frequency of A + T is the same as that of G + C at the third position, then the codon usage preference is likely to be entirely caused by mutation [40]. The A3s/(A3s+T3s) and G3s/(G3s+C3s) of each gene were calculated and used as the ordinate and the abscissa to show the relationship between the two base contents of genes, namely purine (A and G) and pyrimidine (T and C) at the third codon position. The PR2-plots were drawn by Matlab R2016a (https://www.mathworks.com/).

#### ENC-plot (ENC versus GC3s)

The effective codon number (ENC) determines the degree of preference for the unbalanced use of codons. ENC value ranges from 20 (only one codon is used for each amino acid) to 61 (when all synonymous codons are used for each amino acid) and is negatively correlated with codon usage bias. The codon usage pattern across genes was examined by the ENC-plot drawn by Matlab R2016a, which is a plot of ENC versus GC3s. ENC-plot is one of the most widely used measures for judging whether or not organism codon usage is biased through exploring the use of codon bias, and detecting the effect of base content on CUB [36]. The expected ENC values from GC3s (denoted by ‘*S*’) were calculated according to the equation given by Wright [42] and Novembre [43]:

$${ENC}_{expected}\;=\;2+S+\frac{29}{S^{2}+{\left(1-S\right)}^{2}}$$

The genes would be distributed along the standard curve or near the standard curve when codon bias is only affected by mutation, while they would fall below the standard curve if codon bias is influenced by selection and other factors.

#### Translational selection (P2)

The translation option (P2) measures the efficiency of codon-anticodon interactions and provides an indication of translation efficiency as long as information for preferred codon sets is not available. The results of PR2-bias plot can reflect the relationship between purine (A and G) and pyrimidine (T and C) in codon composition. P2 value > 0.5 shows preference for translational selection.

P2 was calculated according to the following equation, where W = A or U, S = C or G, and Y = C or U [2,44–46]:

$$P2\;=\;\frac{WWC+SSU}{WWY+SSY}$$

#### Determination of putative optimal codons

Optimal codon is the preferred codon, which is determined by calculation and sequencing of the ENC values of all genes. Generally speaking, highly expressed genes have a large degree of codon preference and therefore a small ENC value. Low expression genes contain more rare codons and have a larger ENC value. Therefore, the relative level of gene expression is currently generally determined by comparing ENCs. The smaller the ENC value is, the higher the corresponding gene is often expressed. 5% of the sequence data were taken from the upper and lower limit regions of the ordered data set, to establish two high- and low-bias gene datasets. To define optimal codons, we used a T-test to examine the significance of codon usage difference between the two datasets [47]. The RSCU values of the codons from the two databases were compared. If the difference (ΔRSCU) is equal to or greater than 0.08, and codons with a frequency of usage that was significantly higher (P < 0.01) in high-bias genes than that in genes with low bias were defined as the optimal codons [22,48]. SPSS V21.0 was implemented for statistical analysis.

#### RSCU-based cluster analysis

The RSCU value is the ratio between the actual observed values of the codon and the theoretical expectations. It reflects the relative usage preference for the specific composition of codons encoding the same amino acid [49]. If RSCU = 1, codon usage is unbiased; if RSCU > 1, specific codon frequency is higher than other synonymous codons, otherwise, the frequency is low [49].

In the cluster analysis, 4 cotton species and 4 *At*- and *Dt*- subgenomes were clustered according to their RSCU values using the Hierarchical Cluster Analysis (HCA) tool from OmicShare. In the clustering process, each cotton species was used as an object, and the relative use of codon was taken as variable.

## Results and discussion

### Codon base composition and multiple comparisons

**Fig 1:**
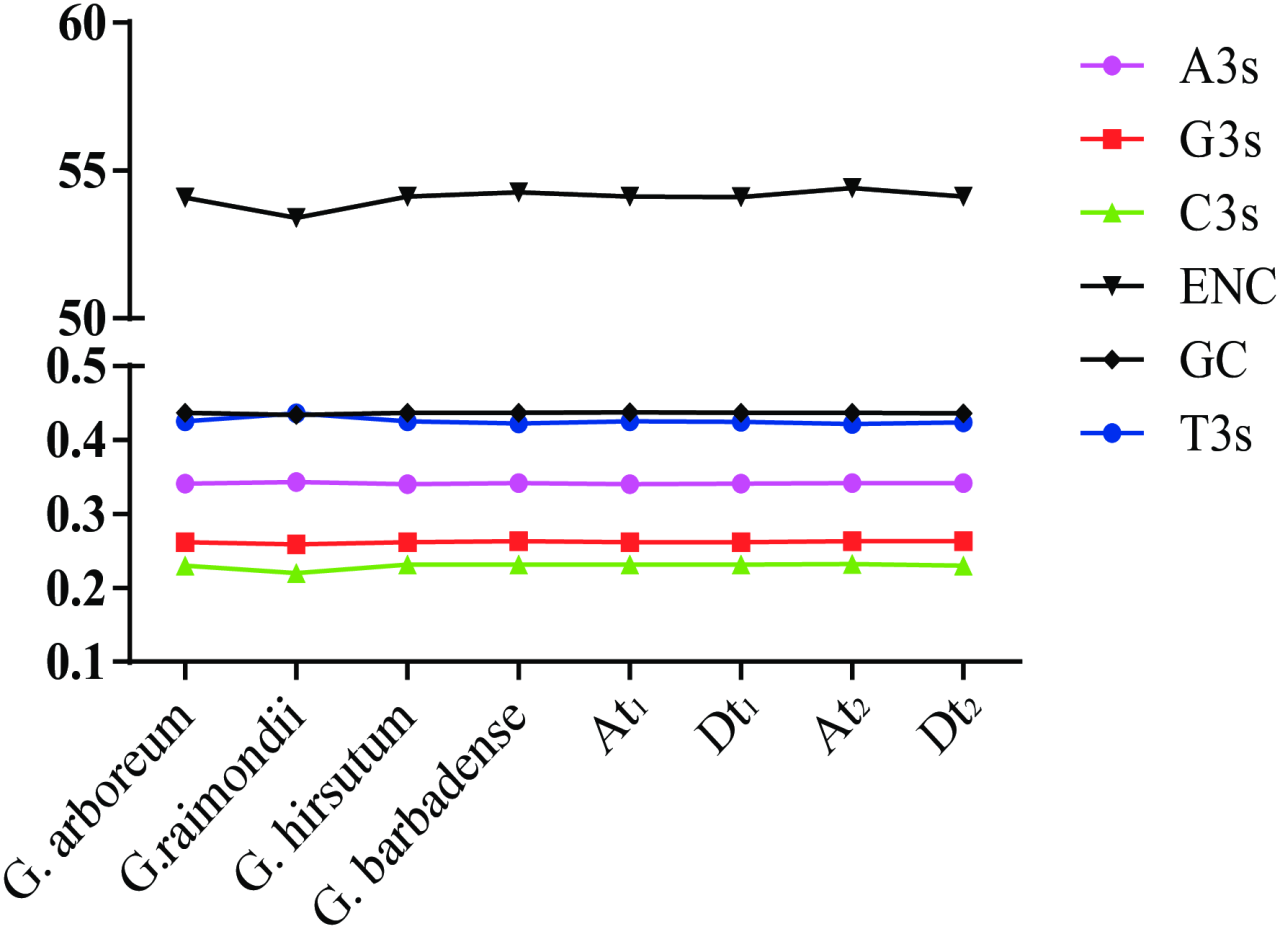
The composition parameters values of codon usage in 4 cotton species and 4 subgenomes.

Firstly, several codon usage parameters were calculated for each cotton species and subgenome taking all their CDSs as a whole and shown in Table 2 and Fig 1. From Table 2 and Fig 1, we can see little difference among all genomes and subgeomes studied except for *G*. *raimondii*, suggesting there are similar codon base compositions at genome and subgenome level. Briefly, the base composition at the third codon position conforms to T > A > G > C. Both the GC3s and GC content were less than 0.5, illustrating that these four cotton species tend to use pyrimidine-rich codons more frequently than purine-rich codons. In *G*. *raimondii*, the T3s was even greater than GC content. The average GC contents are higher than GC3_S_. Such codon base composition preference was also previously reported in upland cotton chloroplast genes whose GC3s (27.38%) and GC (37.89%) were even lower [36]. Totally, these results were consistent with the lower nuclear GC contents in such cotton species [50–53].

**Table 2:** The composition parameters values of codon usage in 4 cotton species and 4 subgenomes.

Species and subgenomes	T3s	A3s	G3s	C3s	GC3s	GC	ENC
*G*. *arboreum*	0.425	0.341	0.262	0.230	0.381	0.437	54.08
*G*. *raimondii*	0.437	0.344	0.259	0.220	0.370	0.434	53.39
*G*. *hirsutum*	0.425	0.341	0.262	0.232	0.382	0.437	54.11
*G*. *barbadense*	0.423	0.342	0.263	0.232	0.383	0.437	54.26
*At*_*1*_	0.425	0.340	0.262	0.231	0.382	0.438	54.11
*Dt*_*1*_	0.425	0.341	0.262	0.232	0.382	0.437	54.10
*At*_*2*_	0.423	0.342	0.264	0.233	0.384	0.437	54.41
*Dt*_*2*_	0.424	0.342	0.263	0.230	0.382	0.436	54.11

**Fig 2:**
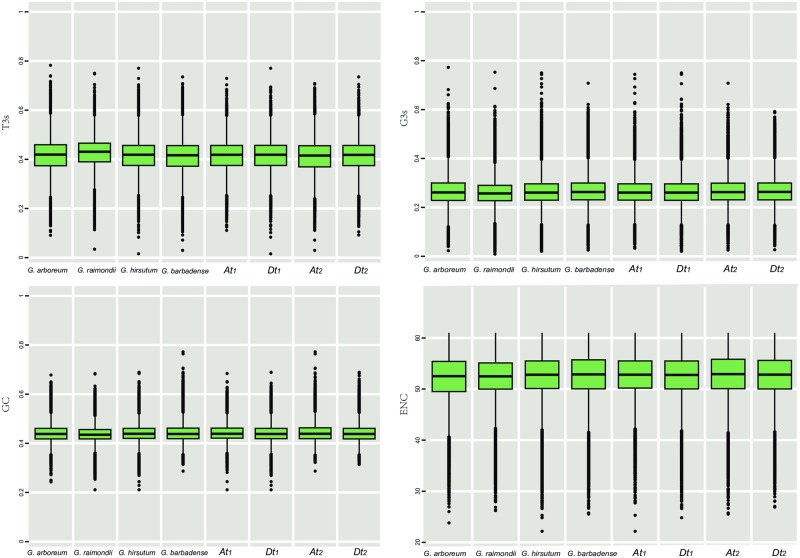
The distribution of T3s, GC3s, GC and ENC of genes in 4 cotton species and 4 subgenomes.

The effective codon number (ENC) values revealed the degree of CUB. ENC is negatively correlated with CUB. According to previous studies [19,42,54], ENC values less than 35 mean high codon preference and ENC values more than 50 reveal general random codon usage. Herein, the average ENC values ranged from 53.39 in *G*. *raimondii* to 54.41 in *At*_*2*_ subgenome (Table 2). The distribution of ENC values of the total genes in each genome or subgenome (Fig 2) revealed that less than 0.5%, and more than 70% of genes had low (< 35) and high (> 50) ENC values respectively in all genomes and subgenomes, indicating weak codon usage bias.

In order to further explore their differences in codon base compositions among these genomes and subgenomes, several indices were selected and calculated for each gene, and distribution analysis (Fig 2) and multiple comparisons (Table 3) were performed. In Fig 2, we got roughly similar distribution patterns of the four SCUB indices among the genomes and subgenomes. A majority of genes distributed relatively concentrated, close to the mean value, and there were some genes with extreme values distributed far away from the mean values of T3s, GC, GC3s, and ENC in two directions.

**Table 3:** Values of T3s(av), G3s(av), GC(av) and ENC(av) of genes in 4 species and 4 subgenomes and their multiple comparisons.

Genomes and subgenomes	T3s_(av)_ * $\bar{x}\pm SD$	G3s_(av)_ * $\bar{x}\pm SD$	GC_(av)_ * $\bar{x}\pm SD$	ENC_(av)_ * $\bar{x}\pm SD$
*G*. *arboretum*	0.4147±0.0677^b^	0.2670±0.0624^b^	0.4413±0.0378^c^	52.06±0.0267^e^
*G*. *raimondii*	0.4252±0.0594^a^	0.2607±0.0540^d^	0.4378±0.0334^d^	52.35±0.0160^d^
*G*. *hirsutum*	0.4133±0.0632^c^	0.2655±0.0592^c^	0.4422±0.0358^bc^	52.47±0.0190^c^
*G*. *barbadense*	0.4116±0.0651^d^	0.2683±0.0580^a^	0.4427±0.0368^b^	52.64±0.0171^ab^
*At*_*1*_	0.4130±0.0627^c^	0.2660±0.0584^bc^	0.4432±0.0349^ab^	52.55±0.0263^bc^
*Dt*_*1*_	0.4136±0.0636^bc^	0.2650±0.0599^c^	0.4412±0.0366^c^	52.40±0.0272^cd^
*At*_*2*_	0.4098±0.0663^e^	0.2682±0.0579^ab^	0.4435±0.0378^a^	52.69±0.0241^a^
*Dt*_*2*_	0.4134±0.0638^bc^	0.2684±0.0582^a^	0.4417±0.0358^c^	52.58±0.0242^b^

*: The “(av)” represents the average of all genes. The multiple comparisons were performed by Duncan's Multiple Range Method.

The various lowercase letters following the data in the same column indicate significant differences at 0.05 level.

However, many significant differences were detected by multiple comparisons (Table 3). The four species genomes showed significant differences in T3s_(av)_, G3s_(av)_ and ENC_(av)_. As for GC_(av)_, significant differences were found between *G*. *raimondii* and other three species, between *G*. *arboreum* and *G*. *barbadense*, while no significant difference was detected between the two tetraploids, and between *G*. *aboreum* and *G*. *hirsutum*.

Comparisons were also performed between *At* or *Dt* subgenomes and their putative progenitor genome respectively. For T3s_(av)_, significant differences were detected among the two *At* subgenomes and *G*. *arboreum* (A_2_), but not among the two *Dt* subgenomes and *G*. *raimondii* (D_5_). With respect to G3s_(av)_, significant differences were detected among the two *Dt* subgenomes and *G*. *raimondii* (D_5_), but not among the two *At* subgenomes and *G*. *arboreum* (A_2_). As for gene average GC content GC_(av)_, the putative donor genome *of G*. *arboreum* (A_2_) had significant lower value than *At*_*1*_ and *At*_*2*_ subgenomes, while *G*. *raimondii* (D_5_) had significant higher value than *Dt*_*1*_ and *Dt*_*2*_ subgenomes. No significant differences were found between *At* and *Dt* subgenomes either in *G*. *hirsutum* or *G*. *barbadense*. For ENC_(av)_, significant differences were detected among the two *At* subgenomes and *G*. *arboreum* (A_2_), and between *G*. *raimondii* and *Dt*_*1*_, but not between *Dt*_*1*_ and *Dt*_*2*_.

We also compared the *At* and *Dt* subenomes in the same genome of the two tetraploids. In *G*. *hirsutum*, significant difference was detected only in GC_(av)_ between *At*_*1*_ and *Dt*_*1*_ subgenomes. Conversely, significant differences were not detected only in GC_(av)_ between *At*_*2*_ and *Dt*_*2*_ subgenomes.

Totally, these four cotton species and the four subgenomes exhibited weak codon usage bias to use pyrimidine-rich codons more frequently than purine-rich codons and there are significant difference in codon base composition and codon usage preference.

### Correlation analysis between codon usage bias indices

Studies have shown that the higher the gene expression level is, the stronger is the preferred use of codon [1–3,6,15,16,49,55–60]. In our study, the codon usage bias results of all cotton species were shown in S1 Table. The correlation between the parameters of 4 cotton species and 4 subgenomes had the same rule, except the correlation between G3s and C3s. There was a negative correlation between G3s and C3s among *G*. *arboreum*, *G*. *barbadense*, *At*_*2*_ and *Dt*_*2*_ subgenomes; and a positive correlation among *G*. *raimondii*, *G*. *hirsutum*, *At*_*1*_ and *Dt*_*1*_ subgenomes.

The results indicated that there was significant negative correlation between the ENC value and T3s, meanwhile, the ENC value was positively correlated with G3s, C3s and GC3s (P <0.01); in addition, T3s had positive correlation with A3s, and negative correlation with G3s, C3s and GC3s. These correlation results indicated that the base content at the third position of the synonymous codons directly affects the degree of codon usage preference. It could be concluded that genes with stronger codon usage bias (with lower ENC value) would have lower G3s, C3s and higher T3s values. These results indicated that the genes of these species and subgenomes preferred to use high expression codons ending with pyrimidines (T/A).

GC12 represents the average GC content of the first and second positions of the codons. A significant correlation between GC12 and GC3 values means that mutational stress is superior to translation selection in the formation of codon usage bias while non-significant correlation between them reveal that translation selection plays dominant role in codon usage preference [55,61–63]. In our study, firstly we took the nuclear genome CDSs of each cotton species and subgenome as a whole and calculated one GC12/GC3 value per cotton species to analyze the correlation coefficients between GC12 and GC3 in 4 cotton species and 4 subgenomes. From the results in Table 4, there was no significant correlation between GC12 and GC3, implying that codon usage bias was influenced primarily by translation selection in these 4 cotton species and 4 subgenomes.

**Table 4:** The correlation coefficients between GC12 and GC3 in 4 cotton species and 4 subgenomes.

	GC1	GC2	GC12
GC2	.827/.969*		
GC12	.984*^/^.992**	.914/992**	
GC3	-.929/-.283	-.610/-.401	-.865/-.344

The digits before and after backslash represent correlation coefficient among 4 cotton species and 4 subgenomes, respectively.

* Correlation was significant at the 0.05 level (2-tailed).

** Correlation was significant at the 0.01 level (2-tailed).

### PR2-bias plot analysis

**Fig 3:**
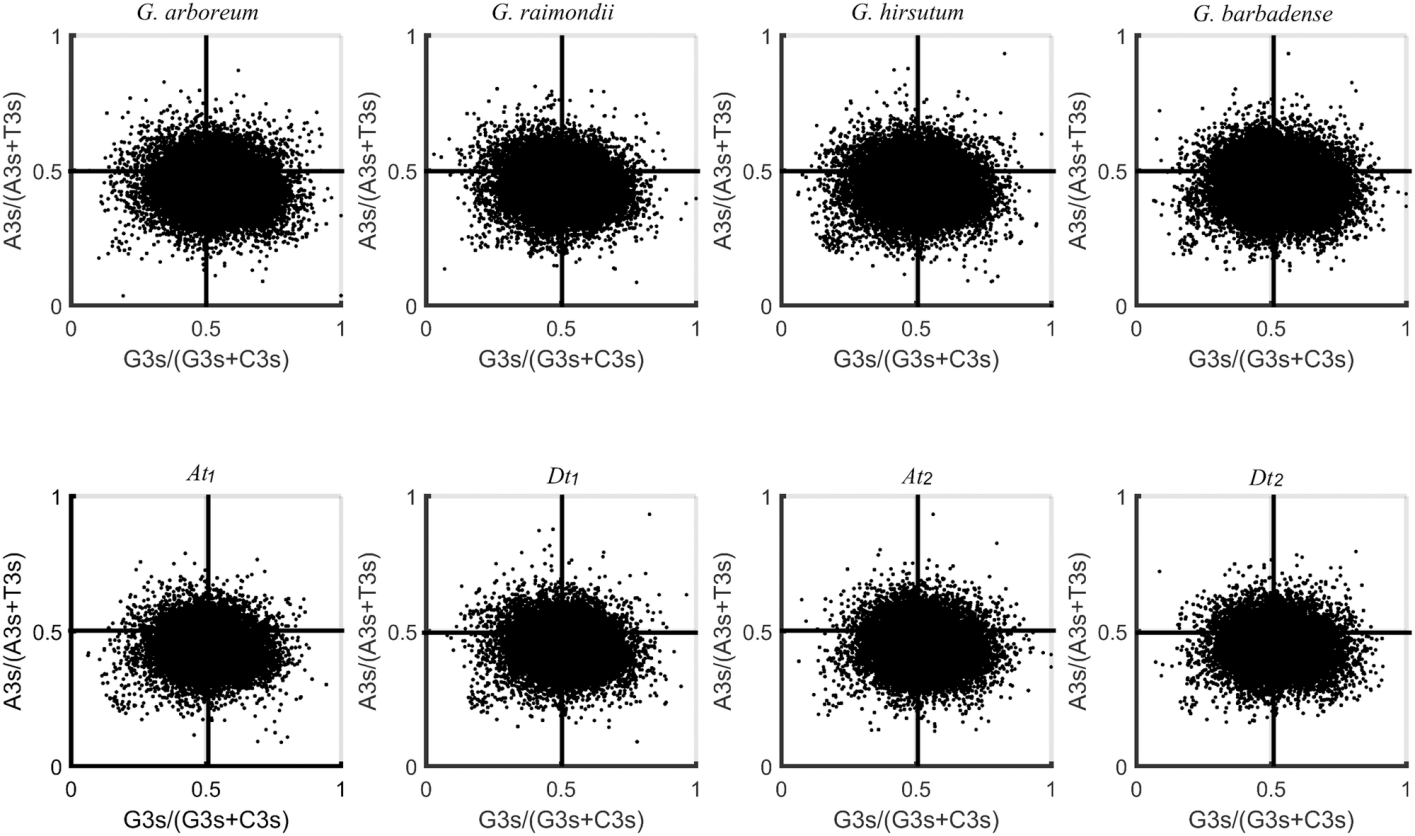
The PR2-bias plots of 4 cotton species and 4 subgenomes.

The PR2-bias plots of the four cotton species and four subgenomes were shown in Fig 3. From Fig 3, it can be seen that along the ordinate, all species genomes and subgenomes presented similar distribution that a majority of genes distributed on the lower left area or the lower right area. However, along the abscissa, there were two types of distributions. A slightly more number of genes of *G*. *arboreum*, *G*. *barbadense* and its two subgenomes distributed on the G > C side than the G < C side while nearly equal amount of genes of *G*. *raimondii*, *G*. *hirsutum* and its two subgenomes distributed on both sides. These results revealed a codon usage imbalance between A + T and G + C at the third base position and indicated that not only the mutation, but also the selection and other factors determined the codon usage patterns in these four cotton species and four subgenomes, and the degree of the third codon position preferences in *G*. *arboreum* and *G*. *barbadense* are slightly different from *G*. *raimondii* and *G*. *hirsutum*. And this was similar to the codon usage bias in chloroplast genome of *G*. *hirsutum* that SCUB was formed under effect of both mutation and selection [36].

### ENC and GC3s scatter plot (ENC-plot)

**Fig 4:**
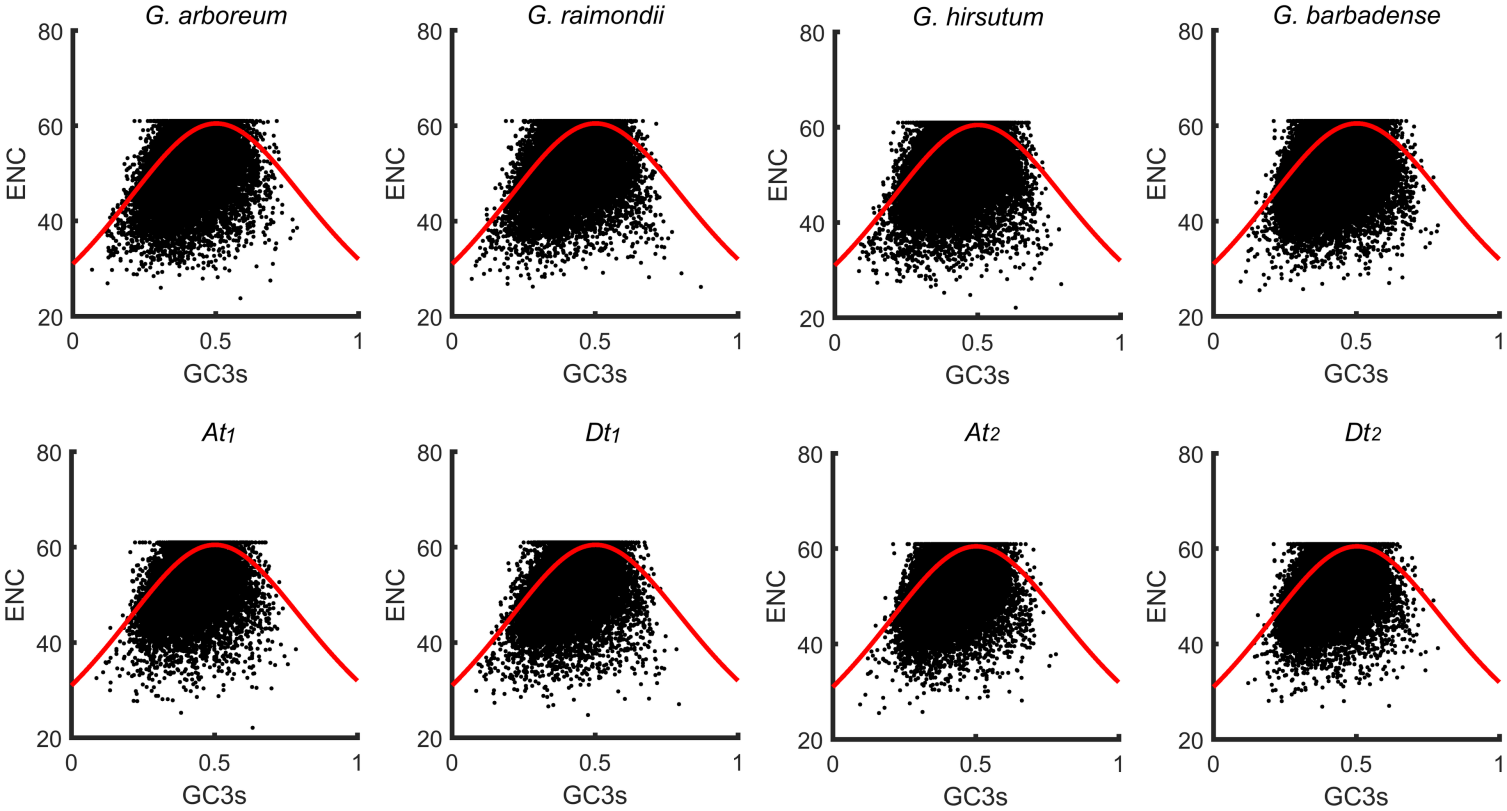
The ENC-plot of 4 cotton species and 4 subgenomes.

Since ENC is constrained by G+C content of the gene, it is often plotted against GC3s of the gene to investigate patterns of codon usage [42,64]. The ENC-plot of CDSs of four cotton species was presented in Fig 4. The solid curve represented the expected position of CDSs whose codon usage was only shaped by the GC3s. Similar ENC-plots are found among all the four cotton species genomes and the four subgenomes of the two tetraploid species (Fig 4). CDSs appeared to cluster around the expected ENC of 30–60. Although a small number of genes distributed in the vicinity of the expected curve, indicated that compositional constraint was the only determinant factor shaping the codon usage pattern. A majority of genes with low ENC values deviated well below the expected curve, indicating that GC3s value was a major determinant of codon usage bias and that other factors independent of nucleotide composition shaped codon usage as well [65]. Consequently, the codon usage pattern of genes in the four cotton genomes and the four subgenomes might be shaped by the combined effects of directional mutation and neutral selection.

### RSCU values analysis and determination of putative optimal codons

The RSCU values of CDSs in 4 cotton species and 4 subgenomes were calculated and shown in S2 Table. The RSCU values of 25 codons were greater than 1 and 31 codons were smaller than 1 in all the cotton species, except AAG (Gly), GUG (Gly), and AAA (Lys). Among them, AAG is a low frequency codon in *G*. *raimondii*, on the contrary, it appeared with high frequency or no preference in other cotton species. In addition, these high frequency codons mostly ended with T (15 of 25) or A (8 of 25) (except UUG and AGG). *At* the same time, most of the low frequency codons ended with C (16 of 31) or G (9 of 31). The above results showed that the RSCU values and such preference of all codons maintained a high degree of unity among all the cotton species.

By comparing the RSCU values from two bias libraries of each cotton species, 19 to 26 optimal codons were determined for each cotton species (Table 5), 15 of them ending with T, 9 ending with A, 2 ending with G. However, although most optimal codons ended in either T or A, codons with T at the third position were detected more frequently. There were 19 common optimal codons in these four cotton species.

**Table 5:** The optimal codons of 4 cotton species and 4 subgenomes.

Codon	*G*. *arboreum*		*G*. *raimondii*		*G*. *hirsutum*		*G*. *barbadense*		*At*_*1*_		*Dt*_*1*_		*At*_*2*_		*Dt*_*2*_	
	High	Low	High	Low	High	Low	High	Low	High	Low	High	Low	High	Low	High	Low
UUU	1.24	1****	1.24	1****	1.21	0.99****	1.22	0.98****	1.18	0.98****	1.23	1****	1.21	0.97****	1.22	1****
UUA	1.04	0.88***	1.1	0.88****	1.07	0.87****	0.99	0.9****	0.98	0.86****	1.16	0.89****			1	0.9****
UUG	1.7	1.41****	1.67	1.4****	1.69	1.41****	1.75	1.3****	1.71	1.41****	1.66	1.41****	1.7	1.23****	1.79	1.38****
CUU	1.46	1.27****	1.54	1.32****	1.49	1.29****					1.49	1.29****				
AUU	1.55	1.18****	1.53	1.22****	1.48	1.2****	1.53	1.18****	1.49	1.2****	1.47	1.21****	1.54	1.18****	1.53	1.19****
GUU	1.79	1.38****	1.91	1.42****	1.86	1.39****	1.86	1.3****	1.88	1.39****	1.85	1.4****	1.84	1.27****	1.88	1.34****
UCU	1.58	1.15****	1.65	1.23****	1.61	1.16****	1.5	1.15****	1.6	1.16****	1.64	1.17****	1.5	1.14****	1.51	1.18****
UCA	1.36	1.15****	1.49	1.17****	1.52	1.14****	1.43	1.16****	1.5	1.14****	1.53	1.14****	1.45	1.16****	1.41	1.15****
CCU	1.61	1.26****	1.63	1.28****	1.53	1.25****					1.53	1.25****				
CCA	1.54	1.21****	1.52	1.18****	1.61	1.19****	1.55	1.22****	1.59	1.17****	1.62	1.2****	1.57	1.25****	1.53	1.19****
ACU	1.59	1.18****	1.59	1.19****	1.53	1.16****	1.54	1.14****	1.55	1.15****	1.51	1.16****	1.53	1.12****	1.56	1.17****
ACA	1.2	1.05***	1.25	1.08****	1.21	1.05****	1.21	1.11****	1.17	1.05****	1.25	1.05****			1.21	1.07****
GCU	1.9	1.38****	1.96	1.42****	1.91	1.4****	1.87	1.34****	1.9	1.39****	1.91	1.39****	1.87	1.29****	1.88	1.38****
GCA					1.16	1.07****			1.15	1.06****					1.17	1.08****
UAU	1.31	1.03****	1.31	1.06****	1.3	1.02****	1.25	1.02****	1.27	1.02****	1.32	1.03****	1.23	1.02****	1.27	1.03****
CAU	1.44	1.14****	1.37	1.15****	1.38	1.14****	1.41	1.12****	1.38	1.13****	1.38	1.16****	1.41	1.1****	1.42	1.16****
CAA	1.36	1.16****	1.32	1.16****	1.37	1.16****	1.33	1.18****	1.34	1.16****	1.4	1.17****	1.31	1.18****	1.35	1.18****
AAU	1.29	1.04****	1.27	1.06****	1.25	1.03****	1.26	1.06****	1.22	1.02****	1.28	1.03****	1.25	1.07****	1.26	1.04****
GAU	1.52	1.27****	1.53	1.28****	1.52	1.26****	1.51	1.25****	1.51	1.25****	1.52	1.26****	1.49	1.25****	1.52	1.25****
GAA			1.19	1.09****	1.19	1.09****					1.21	1.09****				
UGU	1.23	0.98***	1.21	0.99****	1.19	0.96****	1.22	0.99****	1.18	0.96****	1.2	0.97****	1.21	1****	1.23	0.99****
AGU	1.22	0.94****	1.12	0.91****	1.08	0.89****	1.2	0.96****	1.03	0.89****	1.1	0.88****	1.17	0.96****	1.22	0.95****
AGA	2.24	1.36****	2.38	1.4****	2.32	1.32****	2.25	1.34****	2.23	1.31****	2.39	1.33****	2.19	1.32****	2.31	1.36****
AGG	2.01	1.39****	1.83	1.34****	1.87	1.35****	2.06	1.33****	1.9	1.32****	1.84	1.36****	2	1.31****	2.06	1.35****
GGU	1.49	1.14****	1.49	1.13****	1.51	1.14****	1.45	1.14****	1.5	1.13****	1.51	1.15****	1	1.13****	1.43	1.14****
GGA	1.25	1.12****	1.28	1.19****	1.26	1.13****	1.21	1.13****	1.24	1.13****	1.28	1.13****				

*** Correlation is significant at the 0.005 level.

**** Correlation is significant at the 0.001 level.

Compared with the chloroplast genome in *G*. *hirsutum* [36], the optimal codons of the host nuclear genome in the present study were quite different. The number of optimal codons detected in chloroplast genome [36] and nuclear genome in *G*. *hirsutum* herein were 23 and 26 respectively, with 12 shared optimal codons (UUG, AUU, UCU, ACU, GCU, CAA, UGU, AGA, GGU, UUA, CCU and GAA). Most of the rest 11 optimal codons specifically determined in chloroplast genome ended with C (7 of 11) while most of the rest 14 optimal codons specifically determined in nuclear genome end with U (8 of 14).

### Analysis of translational selection (P2) and choice between pyrimidines in the third position of codon

Grosjean et al first noted in the *MS2 phage* genome that there was a bias in the choice between C and U bases in the third position of codon [44,66]. They found that nucleotides at degenerate positions consistently produced moderate-strength codon-anticodon binding energy. If the first two bases of a codon are both A or U, the C at the third position will give a closer to average codon binding energy than U. Similarly, if the first two bases are either C or G, the third base of "right choice" is U because C gives a strong binding energy. Therefore, we can characterize the translational efficiency of a gene by the frequency of "correct selection", called P2 index, between the pyrimidines in codons starting with AA, AU, UA, UU, CC, CG, GC or GG.

First, the values of WWC, SSC, WWU and SSU were calculated according to the RSCU values of the corresponding codons (Table 6). From Table 6, it was seen that both SSU and WWU were higher than SSC and WWC in all species and subgenomes, especially in *G*. *raimondii* with the largest SSU and WWU and the lowest SSC and WWC. That is, the choice between two pyrimidines (U and C) in the third position of codon tends to U. Then the P2 index of species and subgenomes was calculated (Table 6). All species and subgenomes had P2 values more than 0.5, which revealed that translational selection played the dominant role over mutation pressure in the codons’ usage.

**Table 6:** The values of WWC, SSC, WWU, SSU and P2 in 4 cotton species and 4 subgenomes.

Genomes and subgenomes	SSU	WWU	SSC	WWC	P2
*G*. *arboreum*	5.30	4.94	2.40	3.28	0.5389
*G*. *raimondii*	5.44	5.09	2.33	3.11	0.5354
*G*. *hirsutum*	5.31	4.94	2.40	3.29	0.5395
*G*. *barbadense*	5.32	4.94	2.40	3.29	0.5398
*At*_*1*_	5.29	4.94	2.41	3.29	0.5386
*Dt*_*1*_	5.26	4.94	2.40	3.29	0.5381
*At*_*2*_	5.25	4.93	2.43	3.30	0.5374
*Dt*_*2*_	5.26	4.94	2.39	3.28	0.5381

### RSCU-based cluster analysis

**Fig 5:**
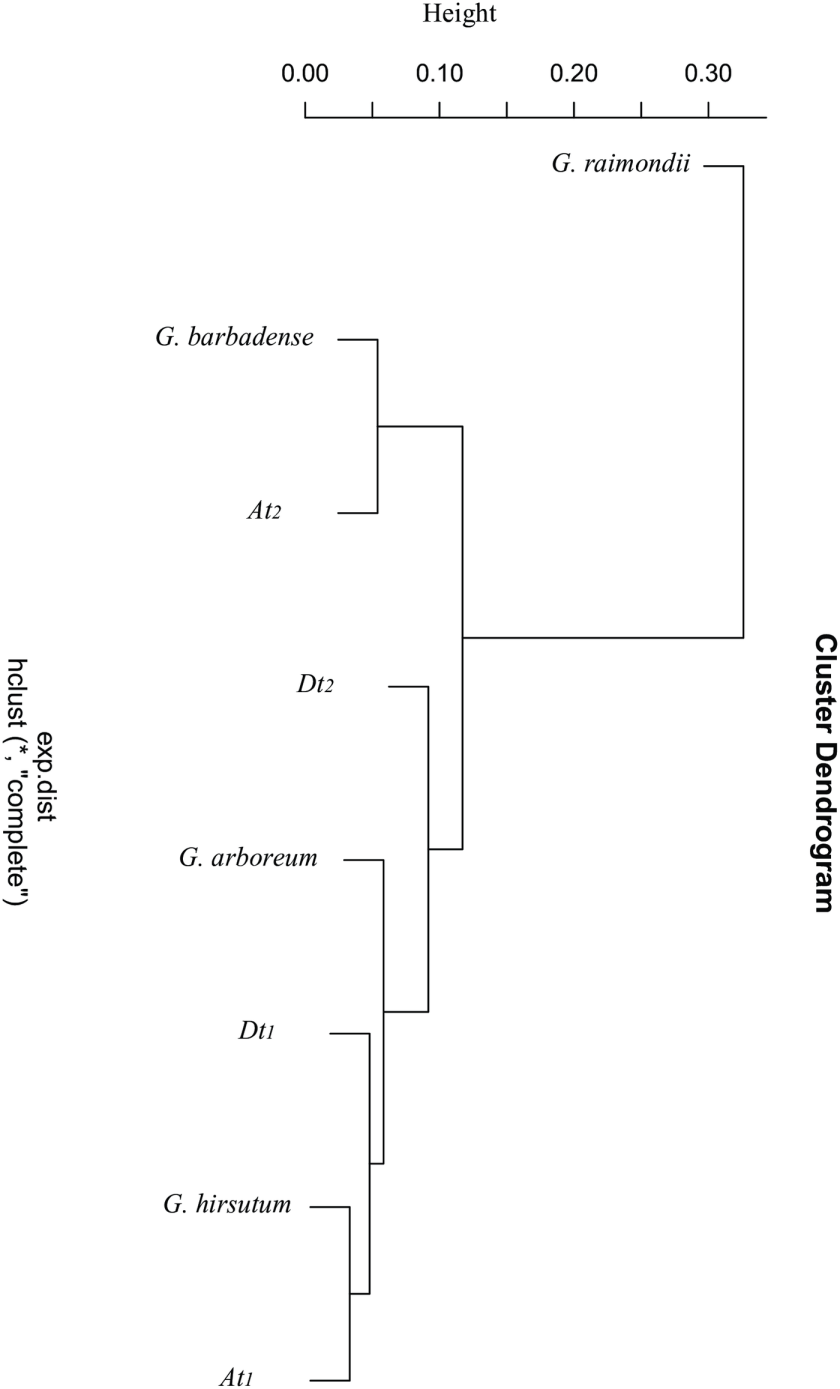
Cluster tree based on the RSCU values of 4 cotton species and 4 subgenomes.

The results of RSCU-based cluster analysis were shown in Fig 5. In Fig 5, the two tetraploids *G*. *hirsutum* and *G*. *barbadense* were grouped into two different clusters. The two *Dt* subgenomes and the D_5_ genome (*G*. *raimondii*) were grouped into different clusters, so as to the two *At* subgenomes and the A_2_ genome (*G*. *arboreum*). These results are quite inconsistent with the widely accepted taxonomic and phylogenetic relationship of these four cotton species [24,26,28,67–70]. The allopolyploid cotton species may have appeared through hybridization and subsequent polyploidization events between the A- and D-subgenome progenitors. The D-genome species *G*. *raimondii* (D_5_) and the A-genome species much like modern *G*. *arboreum* (A_2_) and *G*. *herbaceum* (A_1_) have been supported by molecular methods and other evidence [28,68,71–72] to be the donor species for the *Dt* and *At* subgenome of the tetraploid cotton species, respectively. The monophyly of polyploid Gossypium species was also studied through cluster analysis based on sequences of a 2.8-kb intergenic region from all diploid species belonging to the genome groups from which the polyploid originates [28], in which all the *Dt* subgenomes and D_5_ genome were grouped into one cluster, and all the *At* subgenomes and the A_1_ and A_2_ genomes were grouped into another cluster. The results of the present study indicated that the evolutionary relationship among these four cotton species could not be well reflected by RSCU-based cluster analysis.

## Conclusions

In the present study, codon usage bias patterns and the shaping factors in the four sequenced cotton genomes of *G*. *arboreum* (A_2_), *G*. *raimondii* (D_5_), *G*. *hirsutum* (AD_1_) and *G*. *barbadense* (AD_2_), and the four subgenomes (*At*_*1*_, *Dt*_*1*_, *At*_*2*_, and *Dt*_*2*_) of these two tetraploids were addressed and compared. All these genomes and subgenomes exhibited similar weaker codon usage bias revealed by the results of less (< 0.5%) genes with low (< 35) ENC and more genes (> 70%) with high ENC. Codon composition analysis revealed these species and subgenomes had low GC and GC3, tended to use pyrimidine-rich codons more frequently than purine-rich codons at the third positions of codons and follow the T > A > G > C trend, although there was significant difference in codon composition and codon usage preference among them. Correlation analysis indicated that the base content at the third position of codons affected the degree of codon preference. PR2-bias plot and ENC-plot revealed that not only translation selection but also directional mutation and other factors shaped the CUB. The P2 analysis results, with the non-significant correlation between GC12 and GC3, further revealed that translation selection was the main factor influencing the CUB pattern herein. Through RSCU analysis, 25 high frequency codons preferentially ended with T or A, and 31 low frequency codons preferentially ended with C or G common in these genomes and subgenomes were determined. And 19 to 26 optimal codons were determined, including 19 common ones, for each species and subgenome. The optimal codons preferred to end with A or T. Finally, we concluded that these four cotton genomes had weak CUB, translation selection played dominant role over mutation pressure in codon usage preference in these four cotton species, and *At* and *Dt* subgenomes had similar codon usage patterns with their A- and D-genome progenitors.

## Supporting information

S1 Table The correlation analysis between codon usage bias indices in 4 cotton species and 4 subgenomes. (XLSX)

S2 Table The RSCU values of CDSs in 4 cotton species and 4 subgenomes. (XLSX)
